# Supplementary material for: Fucoxanthin, A Carotenoid Derived from Phaeodactylum tricornutum Exerts Antiproliferative and Antioxidant Activities In Vitro
Source: Antioxidants (Basel). 2019 Jun 19;8(6):183. doi: 10.3390/antiox8060183 (PMC6617271; doi:10.3390/antiox8060183)
Supplement: Supplementary file 1 [file antioxidants-08-00183-s001.pdf]

antioxidants

MDPI

Article

Fucoxanthin, A Carotenoid Derived from *Phaeodactylum tricornutum* Exerts Antiproliferative and Antioxidant Activities In Vitro

Ulrike Neumann [1,†], Felix Derwenskus [2,3,†], Verena Flaiz Flister [1], Ulrike Schmid-Staiger [2], Thomas Hirth [4] and Stephan C. Bischoff [1,*]

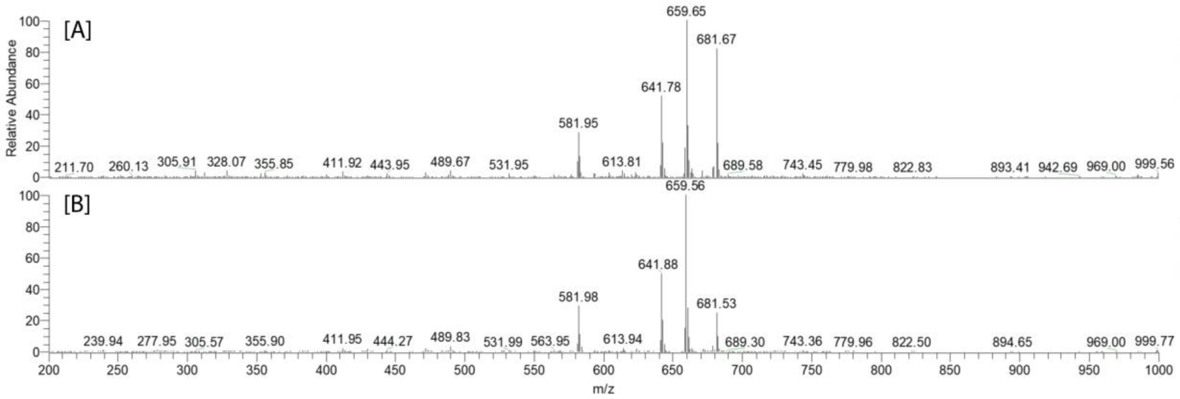

Figure S1. UHPLC-MS spectra of a commercial fucoxanthin standard (**A**) and fucoxanthin derived from the diatom *P. tricornutum* (**B**) showing the specific m/z-values of precursor ions (*m/z* = 659.6 [M+H]+; 681.5 [M+Na]+) of fucoxanthin and related daughter ions (*m/z* = 641,8 and 581,9). Abbreviations: m/z mass-to-charge ratio.
